# Comment on ‘Asian Reference Values for Handgrip Strength, Gait Speed, Five‐Times‐Sit‐to‐Stand Test, Muscle Mass and Calf Circumference’ by Grgic et al.

**DOI:** 10.1002/jcsm.70317

**Published:** 2026-06-17

**Authors:** Wee‐Shiong Lim, Prasert Assantachai, Reshma Merchant, Chang Won Won, Jean Woo, Liang‐Kung Chen, Hidenori Arai

**Affiliations:** ^1^ Department of Geriatric Medicine Institute of Geriatrics and Active Aging, Tan Tock Seng Hospital Singapore; ^2^ Lee Kong Chian School of Medicine Nanyang Technological University Singapore; ^3^ Yong Loo Lin School of Medicine National University of Singapore Singapore; ^4^ Department of Preventive and Social Medicine, Faculty of Medicine Siriraj Hospital Mahidol University Bangkok Thailand; ^5^ Division of Geriatric Medicine, Department of Medicine National University Hospital Singapore; ^6^ Department of Family Medicine, College of Medicine, Kyung Hee University Kyung Hee University Medical Center Seoul Republic of Korea; ^7^ Jockey Club Institute of Ageing The Chinese University of Hong Kong Hong Kong SAR China; ^8^ Center for Geriatrics and Gerontology Taipei Veterans General Hospital Taipei Taiwan; ^9^ Center for Healthy Longevity and Aging Sciences National Yang Ming Chiao Tung University Taipei Taiwan; ^10^ Taipei Municipal Gan‐Dau Hospital Taipei Taiwan; ^11^ National Center for Geriatrics and Gerontology Obu Japan

To the Editor:

We read with great interest the recent article by Grgic et al., which presents pooled pan‐Asian reference values for handgrip strength, gait speed, five‐times‐sit‐to‐stand test (FTSST), skeletal muscle mass and calf circumference [1]. The authors should be commended for assembling an extensive dataset across 20 national cohorts and addressing an important gap in muscle health research in Asia. Notably, the reference values varied across Asian regions, with consistently higher values observed in East and West Asia and lower values in South Asia, underscoring substantial regional heterogeneity.

However, we would like to raise several methodological considerations regarding the derivation and interpretation of these pooled ‘normative’ values. Specifically, we question whether the reported pan‐Asian centile distributions can be interpreted as true normative references, given substantial heterogeneity in measurement protocols, cohort composition and construct definitions across contributing studies.

## 1 Conceptual Considerations: Normative Versus Pooled Distributions

Normative reference values are generally expected to reflect distributions derived from populations that are broadly representative and measured using standardized methodologies [2]. In clinical and epidemiological practice, thresholds for low muscle strength or performance are typically anchored to the lower tail of such distributions (e.g., 10th–20th percentile), particularly when defining physiological deficit [3]. Importantly, such reference populations should also be consistent across the different domains of muscle health to ensure comparability of derived metrics [4].

In this context, it is notable that, due to differential data availability across muscle health measures, distinct subsets of cohorts were used to derive the respective reference distributions. For example, FTSST and skeletal muscle mass values were derived from only four cohorts, whereas calf circumference was derived from just two cohorts. This lack of uniformity in contributing populations introduces an additional layer of heterogeneity that is not explicitly accounted for in the pooled estimates.

Importantly, when contemporary diagnostic thresholds—such as those proposed in the Asian Working Group for Sarcopenia (AWGS) 2025 consensus—are mapped onto the centile distributions reported by Grgic et al., they consistently correspond to mid‐ or upper‐range percentiles rather than the expected lower tail [5]. For example, AWGS thresholds for handgrip strength (< 28 kg for men, < 18 kg for women in older adults) align approximately with the 40th–60th percentile, whereas gait speed thresholds (< 1.0 m/s in those aged ≥ 65 years and < 1.2 m/s in those aged 50–64 years) correspond to substantially higher centiles. Similar patterns are observed for FTSST and calf circumference.

To illustrate this more explicitly, we mapped AWGS 2025 thresholds onto the pooled centile distributions (Table 1). Notably, these thresholds consistently align with mid‐ to upper‐range percentiles across all measures. Even handgrip strength—the most standardized and biologically grounded metric—maps to approximately the 40th–60th percentile (Appendix A), whereas gait speed thresholds extend into the upper distribution, particularly in middle‐aged groups. This pattern is not confined to a single measure but is observed consistently across independent domains of muscle health.

**TABLE 1:** Approximate mapping of AWGS 2025 cutoffs to percentiles in pooled pan‐Asian distributions.

Measure	AWGS 2025 cutoff	Age group	Approximate percentile position^a^	Interpretation
Handgrip strength (men)	< 28 kg	≥ 65 years^b^	~40th–70th percentile	Mid‐distribution; not lower tail
Handgrip strength (women)	< 18 kg	≥ 65 years^b^	~40th–70th percentile	Mid‐distribution; not lower tail
Handgrip strength (men)	< 34 kg	50–64 years	~50th–60th percentile	Upper‐mid distribution
Handgrip strength (women)	< 20 kg	50–64 years	~40th–50th percentile	Upper‐mid distribution
Gait speed	< 1.0 m/s	≥ 65 years^b^	~80th–90th percentile	Upper tail
Gait speed	< 1.2 m/s	50–64 years	~90th–95th percentile	Upper tail
FTSST	≥ 12 s	≥ 65 years^b^	~20th–60th percentile	Mid‐distribution, broad range
FTSST	≥ 10 s	50–64 years	~30th–50th percentile	Mid‐distribution
Calf circumference (men)	< 34 cm	≥ 65 years^b^	~60th–80th percentile	Upper‐mid distribution
Calf circumference (women)	< 33 cm	≥ 65 years^b^	~60th–80th percentile	Upper‐mid distribution

^a^ Percentiles are approximated by mapping AWGS 2025 thresholds to pooled Asian centile distributions reported by Grgic et al. Mappings are intended to provide indicative ranges rather than precise estimates.

^b^ Age range examined: 65–84 years.

This systematic upward shift strongly suggests that the pooled distributions are unlikely to represent true normative references. Rather, they appear to reflect composite distributions that are shifted by underlying cohort heterogeneity and methodological non‐equivalence. If the pooled values were valid normative standards, clinically established thresholds would not be expected to map consistently to central or upper portions of the distribution across multiple independent measures.

## 2 Measurement Heterogeneity and Construct Non‐Equivalence

A major limitation of the pooled analysis lies in the heterogeneity of measurement protocols across cohorts. For handgrip strength, assessments were conducted using different dynamometers (mechanical or electronic), with variation in testing position (seated, standing or lying) and elbow positioning (flexed vs. extended). It was unclear if the average or maximum value was used [6]. Although the authors suggest that such differences introduce relatively small variation (1–4 kg), this magnitude represents a clinically meaningful proportion of diagnostic thresholds and is likely to introduce systematic, rather than random, bias [7]. Gait speed measurements are even more susceptible to protocol variation. Differences in walking distance (e.g., 2.5 m vs. 4 m), start conditions (static vs. dynamic) and timing methodology can produce substantial differences in measured speed. Even modest deviations (e.g., 0.1–0.2 m/s) represent a clinically meaningful proportion of commonly used thresholds and are known to influence associations with adverse outcomes [8].

For skeletal muscle mass, the challenges extend beyond measurement variability to construct validity. The pooling of DXA‐ and BIA‐derived estimates—despite their known non‐equivalence—introduces additional complexity [9]. Furthermore, the use of unadjusted skeletal muscle mass, rather than appendicular lean mass normalized to body size, diverges from established sarcopenia definitions and may confound interpretation by body habitus [4]. Calf circumference is similarly vulnerable to inconsistency. Measurement protocols (e.g., posture, side and anatomical landmark) were not consistently specified across cohorts, despite the known sensitivity of this measure to technique [10]. FTSST performance reflects a composite of strength, balance, coordination and joint function. Variations in chair height, use of upper limbs and test instructions can substantially affect performance, yet standardization across cohorts is unclear [11].

Taken together, these differences suggest that the pooled dataset combines measurements that are not strictly comparable, thereby undermining the assumption of construct equivalence required for generating unified reference distributions.

## 3 Cohort Heterogeneity and Population Comparability

In addition to measurement variability, the contributing cohorts differ substantially in population characteristics. The analysis includes national surveys, regional cohorts and studies with specific disease or ageing focuses. Some cohorts are enriched for older and possibly frailer individuals (e.g., CLHLS and LASI‐DAD), whereas others may represent healthier or more urban populations. Socioeconomic disparities, nutritional status and rural–urban differences across countries further contribute to variability in muscle health metrics.

Importantly, not all cohorts provided sampling weights, necessitating the use of unweighted percentiles. This may result in disproportionate influence of certain cohorts on the pooled distributions. Furthermore, data collection periods spanned two decades (2004–2024), introducing potential secular trends and possible cohort effects that are not explicitly accounted for [12]. Taken together, these findings suggest that the pooled centiles may represent a ‘composite average’ of heterogeneous populations rather than a biologically meaningful normative reference.

## 4 Implications for Interpretation and Application

These considerations have important implications for interpretation. First, the pooled centiles should not be viewed as definitive normative standards for Asian populations, but rather as descriptive summaries of heterogeneous datasets. Second, caution is warranted in using these values to inform diagnostic thresholds or recalibrate existing criteria without outcome‐based validation [5]. Third, future work should prioritize methodological standardization, including harmonized measurement protocols, appropriate normalization of body composition measures and careful selection of reference populations [3, 5].

## 5 Conclusion

The work by Grgic et al. represents an important contribution to muscle health research in Asia. However, the systematic misalignment between pooled centile distributions and established clinical thresholds raises fundamental concerns regarding their validity as normative references. We suggest that these values be interpreted with caution and that future efforts focus on developing standardized, outcome‐validated reference frameworks to support clinical and public health applications.

## Ethics Statement

All authors of this manuscript comply with the guidelines of ethical authorship and publishing in the Journal of Cachexia, Sarcopenia and Muscle.

## Conflicts of Interest

The authors declare no conflicts of interest.

## Patient and Public Involvement

Patients and/or the public were not involved in the design, conduct, reporting or dissemination plans of this research.

## Data Availability

The data that support the findings of this study are available from the corresponding author upon reasonable request.

## Appendix A Illustration Using Handgrip Strength

For the 50–64 age group, the AWGS cutoffs of < 20 kg in women and < 34 kg in men correspond to the 40th–50th percentile and 50th–60th percentile, respectively. Likewise, for the 65–84 age group, the AWGS cutoffs of < 18 kg in women and < 28 kg in men correspond to the 40th–70th percentile in both instances.

**Table:**
